# Learning language with the wrong neural scaffolding: the cost of neural commitment to sounds

**DOI:** 10.3389/fnsys.2013.00085

**Published:** 2013-11-12

**Authors:** Amy S. Finn, Carla L. Hudson Kam, Marc Ettlinger, Jason Vytlacil, Mark D'Esposito

**Affiliations:** ^1^Department of Psychology, University of CaliforniaBerkeley, CA, USA; ^2^Department of Brain and Cognitive Sciences, Massachusetts Institute of TechnologyCambridge, MA, USA; ^3^Department of Linguistics, University of British ColumbiaVancouver, BC, Canada; ^4^Department of Veterans Affairs, Northern California Health Care SystemMartinez, CA, USA; ^5^Helen Wills Neuroscience Institute, University of CaliforniaBerkeley, CA, USA

**Keywords:** language learning, sensitive period, fMRI, plasticity, expertise

## Abstract

Does tuning to one's native language explain the “sensitive period” for language learning? We explore the idea that tuning to (or becoming more selective for) the properties of one's native-language could result in being less open (or plastic) for tuning to the properties of a new language. To explore how this might lead to the sensitive period for grammar learning, we ask if tuning to an earlier-learned aspect of language (sound structure) has an impact on the neural representation of a later-learned aspect (grammar). English-speaking adults learned one of two miniature artificial languages (MALs) over 4 days in the lab. Compared to English, both languages had novel grammar, but only one was comprised of novel sounds. After learning a language, participants were scanned while judging the grammaticality of sentences. Judgments were performed for the newly learned language and English. Learners of the similar-sounds language recruited regions that overlapped more with English. Learners of the distinct-sounds language, however, recruited the Superior Temporal Gyrus (STG) to a greater extent, which was coactive with the Inferior Frontal Gyrus (IFG). Across learners, recruitment of IFG (but not STG) predicted both learning success in tests conducted prior to the scan and grammatical judgment ability during the scan. Data suggest that adults' difficulty learning language, especially grammar, could be due, at least in part, to the neural commitments they have made to the lower level linguistic components of their native language.

## Introduction

Language is an exceedingly complex learned behavioral system. It is well-documented that children ultimately learn this system better than most adults (Snow and Hoefnagel-Höhle, 1978; Birdsong, 1999; Newport et al., 2001; Mayberry and Lock, 2003). However, age-related learning and memory differences usually go in the opposite direction, with young adults consistently outperforming children (Gathercole et al., 2004; Ghetti and Angelini, 2008)^1^. Why is learning language an exception?

One long-posed explanation is that adults' language learning difficulties are the consequence of diminishing neural plasticity (Penfield and Roberts, 1959; Lenneberg, 1967; Pulvermüller and Schumann, 1994). While the mechanisms of plasticity were underspecified in these early proposals, some support for this general idea comes from work showing that cortical sensitivity to different languages in bilinguals is spatially distinct (Whitaker and Ojemann, 1977; Ojemann and Whitaker, 1978; Lucas et al., 2004). These studies applied electric current to cortical regions prior to brain surgery in order to identify (and avoid) language-sensitive regions. Patients also showed more diffuse cortical sensitivity for their second-language (L2) as compared to their native-language. While no causal arguments can be made from these data, the L2 could have a spatially distinct and more diffuse representation because the native-language regions are optimized for (or tuned to) the native-language and therefore cannot process the L2 well. The very process of tuning to the native-language, while beneficial for processing that language, could result in being less open (or plastic) for tuning to the L2.

As compared with this patient work, imaging studies allow the analysis of many more individuals, and therefore permit the exploration of how later—vs. earlier—learned L2s are represented. While both early and late-learned languages are associated with the activation of classic language regions (Klein et al., 1995; Yetkin et al., 1996; Chee et al., 1999; Rüschemeyer et al., 2005, 2006; Indefrey, 2006; Abutalebi, 2008; Consonni et al., 2013), later-learned languages are associated with (1) a greater activation of language regions [especially the left Inferior-Frontal-Gyrus (IFG)] (Dehaene et al., 1997; Chee et al., 2003; Tatsuno and Sakai, 2005; Golestani et al., 2006; Rüschemeyer et al., 2006) and, (2) the involvement of additional (contralateral and subcortical) regions (Klein et al., 1994; Perani et al., 1996; Abutalebi et al., 2013). Likewise, recruitment of the IFG overlaps more for early vs. late bilinguals (Kim et al., 1997) and for more vs. less proficient bilinguals (Perani et al., 1998; Wartenburger et al., 2003; Dodel et al., 2005; Tatsuno and Sakai, 2005; Golestani et al., 2006; Leonard et al., 2011). These studies all suggest that later-learned languages are represented differently, overlapping less with circuitry supporting the native-language.

Neural tuning could explain this. Studies in rats have shown that auditory neurons tune to environmental stimuli (Zhang et al., 2001; Chang and Merzenich, 2003) and that early exposure can lead to more efficient processing of a particular stimulus later on (Insanally et al., 2009). In human infants, behavioral work has shown that a similar tuning process most likely occurs with exposure to native-language phonetics; as infants learn more about the relevant contrasts in their native language they lose the ability (previously held) to distinguish phonetic contrasts not present in their language (Werker et al., 1981). A similar mechanism could be driving age-related differences in the neural representation of language.

Several recent theories of first language acquisition highlight this possibility. These propose that language learning is best viewed as a series of nested sensitive periods; tuning in one area (say to the phonetic categories of one's language) gives rise, in turn, to an ability to learn other aspects of language (Kuhl, 2004; Werker and Tees, 2005). Importantly, these theories suggest that the neural networks dedicated to processing nested aspects of language (i.e., phonetic categories for spoken languages) do not just influence learning at the same level of linguistic knowledge, but also promote (or inhibit) the brain's future ability to learn *other* aspects of language, such as grammar. In other words, the neural networks dedicated to the newly learned languages should differ not just in regions that are directly sensitive to phonetics or grammar, but across the network in terms of how these regions interact with one another.

While such interactions have yet to be explored in the brain, there is some modeling and behavioral evidence for this pattern of nested learning. Modeling work has shown that experience (or the number of training trails) is crucial for tuning: with more training, individual units are more committed (or tuned) to specific functions (see Ramscar et al., 2010). There is also behavioral evidence for this pattern of learning, both for facilitation in L1 acquisition and inhibition in adult L2 acquisition. For instance, Kuhl et al. (2005) found that infants who were good at phonetic contrasts in their native language and poor at irrelevant contrasts (and are therefore more “tuned” to the sound properties of their language) performed better, as compared to those who were less specifically tuned, when measured on other aspects of language processing later-on. And Finn and Hudson Kam (2008) found that adult L2 learners' ability to segment words from running speech via statistical learning was compromised when L1 word formation patterns (phonotactics) conflicted with the L2 word boundaries. Since tuning to novel phones is known to be especially difficult for adults (Golestani and Zatorre, 2004; Zhang et al., 2005; Wong et al., 2007), the nesting hypothesis suggests that this may account for their difficulties with all other aspects of language as well. Moreover, and of particular relevance for the present paper, tuning should influence the neural representation of later-learned languages, both within and across regions, in terms of how they interact with each other.

## Methods

To investigate this, we examine whether non-native L2 phonology (sounds and phonotactics)—defined here as the degree to which it is shared with native language—can affect where L2 *grammar* is processed in the brain. We created two miniature artificial languages (MALs) both with the same syntax but each with different sound systems, which we taught to two different groups of adult learners over the course of 4 days. After the language exposure, participants underwent fMRI scanning while making grammaticality judgments in the MAL they had learned and in English (their native language). Importantly, the shared grammatical structures of the MALs were distinct from English. Crucially, one miniature language was phonologically similar to English (English-Phonology; EP), the other was distinct (Non-English-Phonology; NEP).

If the ideas outlined above are correct, we should observe (1) less overlapping recruitment for the language with distinct phonology (NEP) and English than the EP language and English (Kim et al., 1997), (2) the recruitment of additional regions [including contralateral regions (Golestani and Zatorre, 2004; Perani and Abutalebi, 2005; Klein et al., 2006)] for the NEP vs. the EP language, and (3) more native-like connectivity within the network recruited for the EP language as opposed to the NEP language. Analyses are conducted across the brain and focused especially on the left Inferior Frontal Gyrus (IFG) and left Superior Temporal Gyrus (STG) as both are associated with processing of syntax (Friederici and Kotz, 2003; Musso et al., 2003; Opitz and Friederici, 2007; Herrmann et al., 2012) and speech perception/production (Hickok and Poeppel, 2000).

### Participants

Twenty individuals from the University of California, Berkeley were randomly assigned to learn one of the two languages. Since gender is related to differences in the neural representation of language (Harrington and Farias, 2008), this was balanced across groups, 5 of the 10 NEP leaners were male and 5 of the 10 EP learners were male. Age was also matched (EP: mean: 24.5 yrs, *SD*: 4.99; NEP: mean: 24 yrs, *SD*: 5.27). All participants were right-handed native English speakers with no history of hearing loss and no more than 3 years of classroom based exposure to another language. Participants were excluded if they had any previous exposure to an SOV language or any home-based exposure to a language other than English [since phonetic information can be retained after this kind of experience (Kit-Fong Au et al., 2002)].

### Stimuli

**Figure 1:**
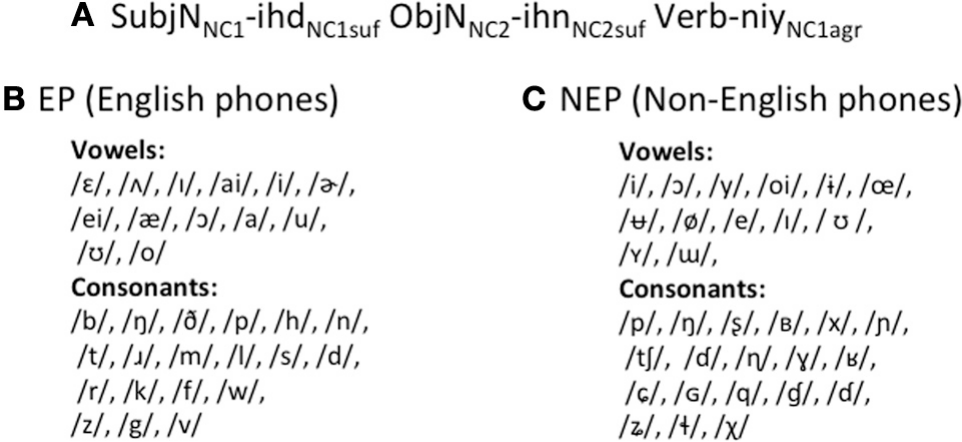
**EP and NEP languages**. EP and NEP languages share the same grammar **(A)**, but have different phonological inventories **(B,C)**.

Both languages comprised 4 transitive verbs, 30 nouns, which were arbitrarily divided into two noun classes, and 4 suffixes. Sentences followed a subject-object-verb word order. All nouns were followed by one of two noun suffixes, which served to indicate noun class membership. There was also subject-verb agreement. The subject agreement suffix depended on the noun class of the subject noun, but was not the same form as the suffix on the noun itself (Figure 1A). Importantly, the two languages have exactly the same grammatical structure as each other, but one which is distinct from English and so requires learning.

Critically, however, the two MALs differ in their phonological inventories. The EP language is comprised of phones that occur regularly in English (Figure 1B). Individual token frequencies were matched to English in both syllable position frequencies, and syllable structure frequencies as closely as possible. For example, if a phone occurs at the beginning of a word 5% of the time in English, this is also true for EP. Likewise, if 20% of English words follow a consonant-vowel-consonant pattern, 20% of EP words do as well^2^. In contrast, the NEP language is comprised mostly of phones that do not occur in English (Figure 1C) drawn from an inventory of phonemes from across the world's languages^3^. To construct words in the NEP language and develop the NEP phoneme inventory, non-native phones were substituted into EP words maintaining major manner and place features. For example, the word for truck in EP, /hIn/, starts with a glottal fricative while the word for truck in NEP, /xyɲ/, starts with a velar fricative; the bilabial voiceless plosive, /p/, is replaced with a bilabial ejective /p'/, and so on. Thus, the NEP has the same number of phonemes as the EP and English.

All stimuli from all three languages (English, EP, and NEP) were recorded in a sound booth by the same male native English-speaker, who is a trained phonetician. To ensure parity of production fluency, the NEP language was practiced several times until speech rate and duration across EP and NEP were approximately equivalent.

The languages were created in conjunction with a small world of objects and actions. Even with the semantic restrictions imposed by the referent world, there are over 3600 possible sentences. This creates a wide scope for testing participants using novel sentences.

### Tests

There were 4 tests—vocabulary, verb agreement, noun class, and word order. Each of these tests was administered at various points during training. Here we present results from the final tests (end point) since that was integral to the design of this study^4^. To test vocabulary, participants viewed a picture, heard three possible labels for that picture and indicated which of the three labels they thought matched the picture with a button press. Verb agreement, noun class and word order were also tested. The tests of verb agreement and noun class were forced choice; learners were asked to indicate which of two sentences sounded like a better sentence in the language they just learned. For verb agreement, they chose between a correct subject-verb pairing and an incorrect pairing with every other aspect of the sentences being equivalent (and correct). For noun-class, they chose between a sentence with a correct noun class suffix and an incorrect noun class suffix; everything else was equal. The word order test was also forced choice; individuals were presented with a scene and heard two possible sentences that could correspond to that scene. One sentence followed the correct subject-object-verb word order and one flipped this arrangement having object-subject-verb word order.

### Procedure

Learning occurred over the course of 4 days and the fMRI scan occurred on the 5th day. To learn, participants watched a series of short scenes on the computer, listened to their corresponding sentences, and repeated the sentences out loud. In order to better mimic naturalistic language learning (as opposed to classroom L2 learning) learners were not given any direct feedback during this training (Hudson Kam and Newport, 2005, 2009). Days 1–3 each consisted of one 90-minute session during which the 57 scenes (and their corresponding MAL sentences) that comprised the stimulus set were repeated three times.

Because we know that difficulty of processing and time on task can drive differences in the blood oxygen level dependent (BOLD) response (Whitaker and Ojemann, 1977; Huettel et al., 2009) and because language proficiency impacts neural representation (Perani et al., 1998), we felt that is was important to match participants' learning-levels (and not necessarily the amount of exposure to the language) prior to participation in the scan. To ensure no differences, participants were tested on all measures at the end of day 3. If after day 3, performance was below 75% on any test, participants were given the full 90-minute exposure on day 4 (the 57 scenes presented three times). If performance was above 75% on all measures, participants were given only 30 min of exposure on day 4 (the 57 scenes were presented only once). This design allowed us to control proficiency prior to the scan, allowing the direct comparison of neural responses across the languages even though the NEP should be harder to learn. Accordingly, four NEP and two EP learners received the 90-minute exposure on day 4, while all other leaners received 30 min of exposure on day 4.

Neural recruitment was probed on day 5 while individuals determined whether a sentence was grammatical or not in alternating blocks of English or the MAL they learned. Blocks were counterbalanced across participants and conditions; half of the scans began with English and the other half began in the MAL they learned. These were presented in blocks so that learners were not required to switch between languages when making grammaticality judgments. This task was chosen in order to engage regions targeting grammatical processing, and not phonology (at least not directly). For each language, 15% of the items were not grammatical. This percentage was chosen to maximize the number of grammatical trials that can be used for data analysis, while having enough ungrammatical items to hold listeners' attention. Ungrammatical English items were modeled after Johnson and Newport (1989). Half of the ungrammatical MAL items were verb agreement errors and the other half were noun class errors. In this event related design, each sentence was presented over noise-cancelling earphones for 4 s, after which participants had 2 s to indicate their response. Sentences across the three languages—English, EP, and NEP—were matched for length. Finally, there was a jittered rest period prior to the next trial (from 2 to 8 s mean length: 5 s). Each trial lasted an average of 11 s; there were 160 trials of each condition, split into 4 runs of 80 trials each.

Functional MRI data were acquired on a Siemens MAGNETOM Trio 3T MR Scanner 291 at the Henry H. Wheeler, Jr. Brain Imaging Center at the University of California, Berkeley. Anatomical images consisted of 160 slices acquired using a T1-weighted MP-RAGE protocol (*TR* = 2300 ms, *TE* = 2.98 ms, *FOV* = 256 mm, matrix size = 256 × 256, 294, voxel size 1 × 1 × 1 mm). Functional images consisted of 27 slices acquired with a continuous gradient echoplanar imaging protocol (*TR* = 2000 ms, *TE* = 32 ms, *FOV* = 1380 mm, matrix size = 128 × 128, voxel size 1.8 × 1.8 × 3.5 mm).

### fMRI analysis

Functional MRI data processing, analysis were completed using a Statistical Parametric Mapping program [SPM5 (Friston et al., 1995)]. Temporal sync interpolation was used to correct for between-slice timing differences. Motion correction was accomplished using a six-parameter rigid-body transformation algorithm, and data were spatially smoothed using 8 mm FWHM Gaussian kernel. A statistical parametric map was calculated for each participant based on linear combinations of the covariates modeling each task period (listening and response for English and the newly learned language separately; correct and incorrect trials were modeled separately and only correct trials were included in the final analyses). These individual results were then combined into a group analysis. All data presented refer to the listening (and not response) phase of the experiment.

Whole brain conjunction analyses was completed using SPM5, following the minimum statistic, conjunction null method in which all of the comparisons in the conjunction must be individually significant (Nichols et al., 2005). In all cases, the conjunction was conducted for the contrasts (1) English > implicit baseline, and (2) new language (EP or NEP) > implicit baseline. Regions of interest (ROI) were created for the left IFG [Broca's region (Amunts et al., 1999)], the left STG (Morosan et al., 2001), and anterior and posterior regions of the left Angular Gyrus [AGa and AGp (Caspers et al., 2006)] using the SPM Anatomy Toolbox (version 1.6; Simon Eickhoff). The number of overlapping voxels (from the conjunction analysis) were counted within these masks for each individual (normalized space). Voxels reaching a range of thresholds (from *t* = 3 to *t* = 5.5) were identified.

In addition, the mean contrast values for processing in the new language (EP or NEP vs. implicit baseline) were extracted from these ROIs (in normalized space) using MarsBar (Brett et al., 2002) and correlated with behavior. Behavioral regressors (learning scores) were included in the second level analysis in order to identify regions—across the brain—most related to behavior. To measure functional connectivity, the magnitude of the task-related BOLD response was estimated separately for each of the experimental trials, yielding a set of beta values for each condition for every voxel in the brain (beta series). The extent to which two brain voxels interact during a task condition is quantified by the extent to which their respective beta series from that condition are correlated (Rissman et al., 2004).

## Results

**Figure 2:**
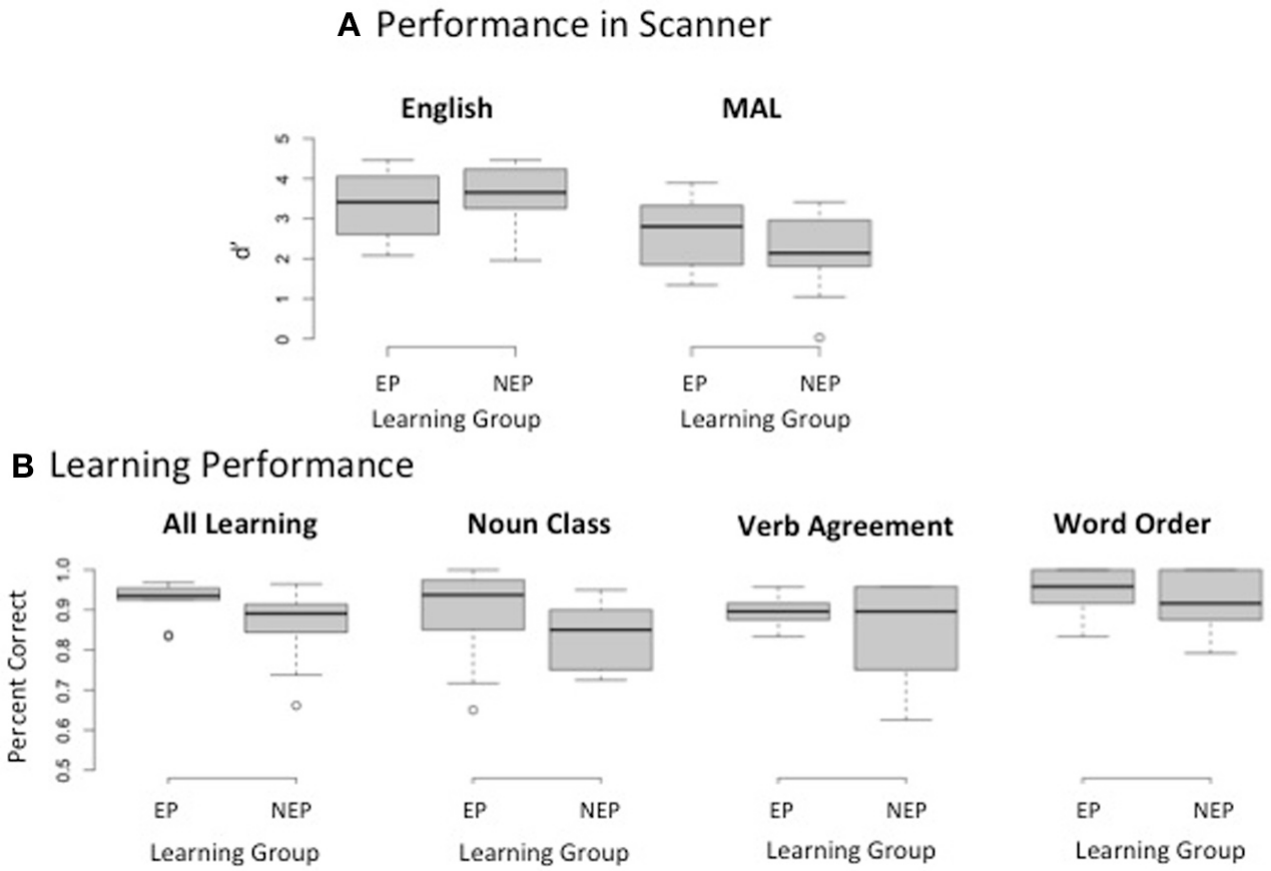
**Behavioral Performance**. Box plots depict the median (middle line), upper quartile (top of box), lower quartile (bottom of box), maximum value (top whisker, excluding outliers), and minimum value (bottom whisker, excluding outliers); outliers are depicted as circles. Discrimination sensitivity (d') does not differ for making grammaticality judgments in either English or the miniature artificial language (MAL) that is learned **(A)** Test performance is also matched prior to entering the scanner on an aggregate measure of learning (overall performance) and each grammatical sub-test (noun class, verb agreement, and word order) **(B)**.

Due to technical errors during data collection, behavioral data during the scan is missing from one individual (an NEP learner). As expected, repeated measures analyses of variance (ANOVAs) reveal a main effect of language such that performance was better [discrimination sensitivity (d′): *F*_(1, 17)_ = 23.130, *p* < 0.001] and faster [*F*_(1, 17)_ = 5.215, *p* = 0.036] for English (mean reaction time from sentence onset = 4392 ms, *SD* = 514) as compared with the MAL (mean reaction time from sentence onset = 4715 ms, *SD* = 317). There was no main effect of learning group [d′: *F*_(1, 17)_ = 0.014, *p* = 0.907; reaction time: *F*_(1, 17)_ = 0.198, *p* = 0.662] and no group by language interaction [d′: *F*_(1, 17)_ = 1.358, *p* = 0.260; reaction time: *F*_(1, 17)_ = 0.127, *p* = 0.725; EP reaction time: mean = 4721 ms, *SD* = 426; NEP reaction time: mean = 4709 ms, *SD* = 146]. Thus, grammaticality judgments did not differ across groups for either English or MAL during the scan (Figure 2A). Likewise, performance across groups was matched prior to the scan overall [average performance on all tests on all test days: *t*_(18)_ = 1.79, *p* = 0.090] and on each grammatical test (average performance on both days tested): noun class *t*_(18)_ = 1.418, *p* = 0.173, verb agreement *t*_(18)_ = 0.916, *p* = 0.372, word order *t*_(18)_ = 0.551, *p* = 0.588; Figure 2B^5^.

**Figure 3:**
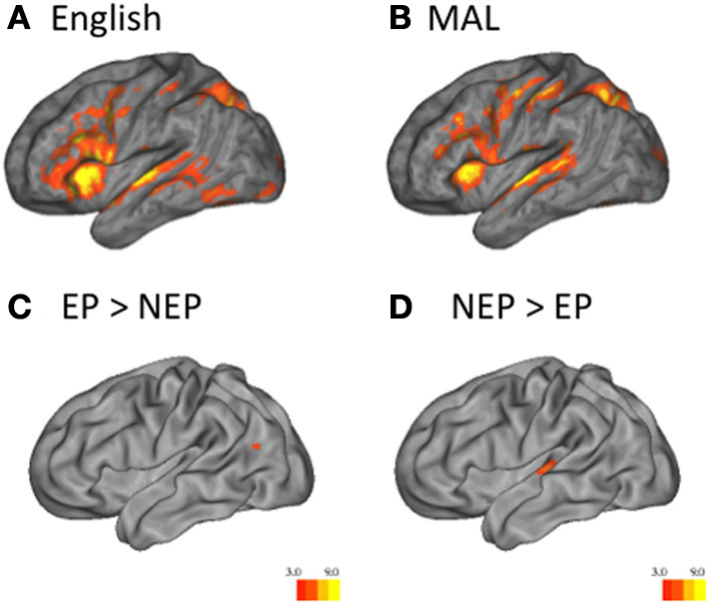
**Univariate Analysis**. One sample *t*-tests reveal that English (vs. implicit baseline) across groups **(A)** and MAL (vs. implicit baseline) across groups **(B)** are associated with the recruitment of classic language regions. Two sample *t*-tests reveal that EP learners recruit the left temporo-parietal region more than NEP learners (EP > NEP) **(C)**, while NEP learners recruit the superior-temporal gyrus more than EP learners (NEP > EP) **(D)** Heat maps indicate the *t*-statistic.

NEP and EP learners both recruited regions known to be critical for language processing while performing grammaticality judgments in English and the MAL they learned (Figures 3A,B; Table 1); all contrasts reported are during the listening period. One sample *t*-tests reveal that regions recruited by both groups for the newly learned language (vs. implicit baseline) include the left IFG (including Broca's region) the Insula (bilaterally) the STG [bilaterally; including posterior language regions, and the Angular Gyrus (Figures 3A,B; Table 1)].

**Table 1:** **Univariate activity during new language processing**.

**MAL > Baseline, all learners (*n* = 20)**							
			**Coordinates (at peak)**				
**Lobe**	**Activation region**	**Hemisphere/Brodmann area**	***x***	***y***	***z***	***t*-score**	***P*-value**
**EP LEARNERS, *n* = 10**							
Frontal	Middle frontal gyrus	L46	−48	22	28	8.94	0.00005
	Middle frontal gyrus	L9	−44	4	40	11.2	0.00005
	Inferior frontal gyrus	L44	−50	6	12	9.8	0.00005
	Precentral gyrus	L4	−46	−8	50	13.03	0.00005
	Insula	L	−32	24	12	9.19	0.00005
	Medial frontal gyrus	L32	−14	22	36	6.73	0.0001
	Superior frontal gyrus	R10	32	48	28	8.33	0.00005
	Anterior cingulate gyrus	R32	12	32	28	11.48	0.00005
	Insula	R	36	28	4	7.32	0.00005
	Superior temporal gyrus	R38	52	16	−8	11.72	0.00005
Temporal	Superior temporal gyrus	R22	62	−20	−2	7.08	0.00005
	Superior temporal gyrus	L42	−54	−36	14	10.05	0.00005
	Transverse temporal gyrus	L41	−64	−16	10	6.39	0.0001
Parietal	Precuneus	R7	10	−74	42	10.95	0.00005
Occipital	Lingual gyrus	R17	−2	−92	−4	9.24	0.00005
	Lingual gyrus	L17	−8	−60	−4	11.58	0.00005
Other	Lentiform nucleus	L	−24	20	−2	15.59	0.00005
	Lentiform nucleus	R	20	16	−6	10.52	0.00005
	Midbrain	R	8	−18	−14	10.21	0.00005
	Midbrain	R	8	−18	−14	10.21	0.00005
**NEP LEARNERS, *n* = 10**							
Frontal	Inferior frontal gyrus	L45	−26	36	8	7.97	0.00005
	Middle frontal gyrus	L46	−46	26	30	7.93	0.00005
	Precentral gyrus	L6	−58	2	30	7.97	0.00005
	Precentral gyrus	L4	−32	−28	58	10.14	0.00005
	Insula	L	−32	18	10	7.17	0.00005
	Superior frontal gyrus	L6	−4	6	60	15.35	0.00005
	Middle frontal gyrus	R46	42	36	30	8.35	0.00005
	Middle frontal gyrus	R9	52	26	40	6.46	0.0005
	Inferior frontal gyrus	R45	38	22	8	7.32	0.00005
	Superior frontal gyrus	R8	2	16	58	7.75	0.00005
	Insula	R	34	22	6	9.16	0.00005
	Superior temporal gyrus	L41	−54	−30	8	12.44	0.00005
Temporal	Superior temporal gyrus	R41	48	−32	10	8.45	0.00005
	Superior temporal gyrus	R42	60	−14	12	9.46	0.00005
	Superior parietal lobule	L7	−28	−66	48	8.23	0.00005
Parietal	Postcentral gyrus	L1	−52	−26	56	8.00	0.0001
	Inferior parietal lobule	R40	40	−48	44	8.35	0.00005
	Middle occipital gyrus	R19	28	−96	14	12.24	0.00005
Occipital	Cuneus	L18	6	−76	12	8.07	0.00005

In this and all other table presenting univariate data, regions are listed where period-specific parameter estimates were significantly greater than baseline (with a t statistic of 3 with a minimum contiguous cluster size to 10 voxels) across scan times.

Across MALs, important differences were observed. Independent sample *t*-tests reveal that EP learners recruit posterior language regions to a greater extent (left temporo-parietal region; EP > NEP; Figure 3C; Table 2), while NEP learners recruit bilateral superior temporal gyrus (STG) more than EP learners (NEP > EP; Figure 3D; Table 2; see Tables 3 and 4 for differences between EP/NEP and English).

**Table 2:** **Univariate Across language comparisons**.

**NEP vs. EP (*n* = 20)**							
			**Coordinates (at peak)**				
**Lobe**	**Activation region**	**Hemisphere/Brodmann area**	***x***	***y***	***z***	***t*-score**	***P*-value**
**NEP > EP**							
Temporal	Superior temporal gyrus	L41, 13, 22	−50	−24	8	5.18	0.001
	Superior temporal gyrus	R42, 41, 22	62	−14	12	3.62	0.001
**EP > NEP**							
Parietal temporal	Middle temporal and angular gyrus	L39	−38	−64	24	4.03	0.001

**Table 3:** **Univariate data: EP and English**.

**EP vs. English, (*n* = 20)**							
			**Coordinates (at peak)**				
**Lobe**	**Activation region**	**Hemisphere/Brodmann area**	***x***	***Y***	***z***	***t*-score**	***P*-value**
**EP > ENGLISH, *n* = 10**							
Frontal	Inferior frontal gyrus	L44	−56	4	22	5.87	0.001
	Superior frontal gyrus	R32	16	16	50	7.08	0.0005
	Middle frontal gyrus	R9	48	8	32	6.77	0.0005
Temporal	Superior temporal gyrus	R42	64	−22	6	6.00	0.0005
Parietal	Superior parietal lobule	L7	−28	−64	50	7.33	0.0005
	Superior parietal lobule	R7	30	−62	58	5.20	0.001
Other	Lentiform nucleus	L	−14	4	4	5.68	0.001
**NEP > ENGLISH, *n* = 10**							
Frontal	Inferior frontal gyrus	L47	−46	40	−10	6.21	0.0005
	Superior frontal gyrus	L9	−20	42	44	6.38	0.0005
	Superior frontal gyrus	L9	−8	54	40	6.84	0.0005
	Inferior frontal gyrus	R47	54	40	−4	4.51	0.001
Temporal	Hippocampus	R	30	−6	−20	5.94	0.0005
	Parahippocampal gyrus	L	−20	−2	−20	5.34	0.001
	Middle temporal gyrus	L21	−56	−18	−22	6.03	0.001
Parietal	Angular gyrus	L39	−54	−66	38	5.45	0.001

**Table 4:** **Univariate data: NEP and English**.

**NEP vs. English, *n* = 20**							
			**Coordinates (at peak)**				
**Lobe**	**Activation region**	**Hemisphere/Brodmann area**	***x***	***Y***	***z***	***t*-score**	***P*-value**
**NEP > ENGLISH, *n* = 10**							
Frontal	Precentral gyrus	R6	36	−6	46	7.47	0.0005
	Middle frontal gyrus	R6	30	−12	66	5.81	0.001
	Insula	R	28	24	12	5.95	0.001
	Precentral gyrus	L6	−30	−14	58	5.56	0.001
	Medial frontal gyrus	L6	−6	2	64	4.93	0.001
Temporal	Superior temporal gyrus	L41, 22, 42	−50	−28	8	7.81	0.0005
	Superior temporal gyrus	L41, 22, 42, 13	68	−20	8	7.38	0.0005
Parietal	Inferior parietal lobule	L40	−30	−52	56	4.58	0.001
Occipital	Cuneus	L18	−2	−84	4	6.05	0.001
**ENGLISH > NEP, *n* = 10**							
Frontal	Inferior frontal gyrus	L47, 45	−30	28	−18	8.27	0.0005
	Middle frontal gyrus	L8	−40	16	54	5.55	0.001
	Superior frontal gyrus	L8	−6	44	52	5.83	0.001
	Middle frontal gyrus	R47	34	40	−10	7.31	0.001
Temporal	Angular gyrus	L39	−38	−60	26	7.21	0.0005
	Parahippocampal gyrus	L20	−34	−36	−22	4.51	0.001
Parietal	Precuneus	L7	−8	−46	46	5.43	0.001

**Figure 4:**
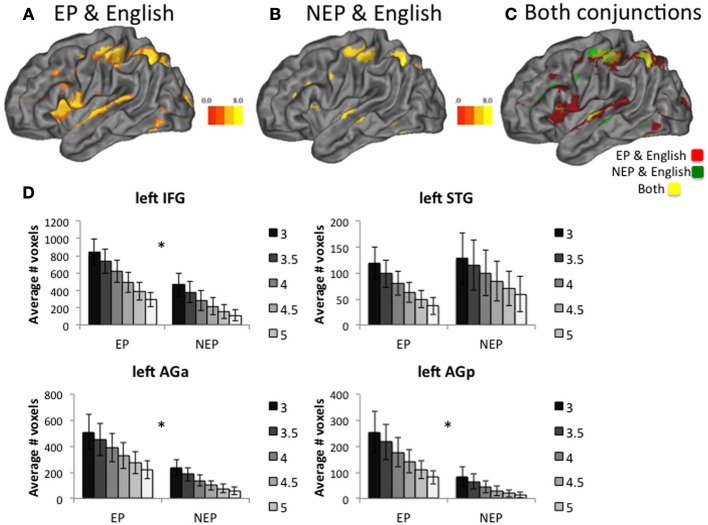
**Conjunction Analysis**. Learners of both languages recruit many overlapping voxels with English **(A,B)**. Overlaying both conjunctions shows differences in the EP and English conjunction (red) and NEP and English conjunction (green) as well as shared regions (yellow) **(C)**. Group *t*-tests reveal that the number of jointly recruited voxels (new language and English) differ across groups in the left Inferior Frontal Gyrus (IFG), the left anterior Angular Gyrus (AGa) for multiple different *t*-statistic thresholds (*t* = 3 through 5), and the left posterior Angular Gyrus (AGp), but not in the left Superior Temporal Gyrus (STG) **(D)**. In all cases, error bars reflect standard error of the mean. ^*^indicates a significant difference at *p* < 0.05.

In the next set of analyses, we use overlap and connectivity methods to explore which recruitment profile (EP vs. NEP) is more similar to English, participants' native language. First, if experience-driven neural tuning contributes to sensitive period phenomena, we should observe less overlapping recruitment for the language with distinct phonology (NEP) and English than EP and English. Both EP and NEP recruitment overlaps with English in the IFG, AG, and STG (along with other regions including the Basal Ganglia; Table 5; Figures 4A,B). To investigate differences across the groups of learners, we counted the number of voxels that were jointly active for English and the new language (EP or NEP; Figure 4C) in the left IFG, left STG, and left AG (posterior and anterior) at multiple different thresholds (*t* = 3, 3.5, 4, 4.5, 5, and 5.5; Figure 4D). We then compared the means of these values across groups using independent samples *t*-tests (Table 6), and found that EP learners have more overlapping recruitment (of the language they learned and English) than NEP learners in the left IFG and AG (both anterior and posterior regions), but not in the left STG (Table 6).

**Table 5:** **Conjunction Analyses**.

**EP and English; NEP and English**							
			**Coordinates (at peak)**				
**Lobe**	**Activation region**	**Hemisphere/Brodmann area**	***x***	***Y***	***z***	***t*-score**	***P*-value**
**NEP and ENGLISH, *n* = 10**							
Frontal	Middle frontal gyrus	L, 46, 9	−50	22	30	6.09	1e-06
	Inferior frontal gyrus	L, 44	−52	6	10	6.62	1e-08
	Inferior frontal gyrus	R, 47, 45	42	20	−4	5.93	1e-06
	Medial frontal gyrus	R, 32, 6	6	14	54	6.59	1e-07
Temporal	Superior temporal gyrus	L, 22	−60	0	−4	5.6	1e-06
	Superior temporal gyrus	R, 22, 38	54	16	−6	7.28	1e-08
Parietal	Postcentral gyrus	L, 40	−44	−34	54	7.15	1e-08
	Superior parietal lobule	L, 7	−28	−66	54	6.36	1e-07
	Superior parietal lobule	R, 7	28	−56	46	8.40	1e-09
	Precuneus	R, 7	16	−79	44	6.19	1e-06
Occipital	lingual gyrus	L, 17	−4	−94	−4	6.0	1e-06
	lingual gyrus	R, 17, 18	14	−90	−10	5.63	1e-06
Other	lentiform nucleus	L	−20	6	4	8.24	1e-09
	lentiform nucleus	R	16	4	8	7.71	1e-07
	Cerebellum (Culmen)	L and R	6	−64	−14	7.21	1e-07
**NEP and ENGLISH, *n* = 10**							
Frontal	Inferior frontal gyrus	L, 45	−30	34	12	6.20	1e-06
	Precentral gyrus	L, 9	−56	4	30	5.25	1e-06
	Superior frontal gyrus	L, 6	−6	4	58	5.89	1e-06
	Middle frontal gyrus	R, 46	32	38	20	6.17	1e-06
Temporal	Superior temporal gyrus	L, 22	−62	−10	4	6.47	1e-06
Parietal	Pre and postcentral gyrus	L, 40, 4	−48	−34	52	5.22	1e-06
	Superior parietal lobule	L, 7, 40	−32	−62	50	6.86	1e-06
Other	Insula	R	36	22	6	6.07	1e-06
	lentiform nucleus	L	−20	0	14	5.77	1e-06
Occipital	Middle occipital gyrus	R, 19	26	−96	14	6.17	1e-06
	lingual gyrus	L, 18	−22	−84	−4	5.68	1e-06

Regions are listed where conjunctions were significant after correcting for multiple comparisons (FDR, *p* < 0.01).

**Table 6:** **Conjunction group differences**.

***T*-test average number of voxels activated**			
**by each contrast (English and MAL)**			
**Region**	**Conjunction statistic**	**Group difference**	***P*-value**
	**threshold**	**(*t*-test)**	
Left IFG	3.0	1.863	0.0395
	3.5	1.878	0.0385
	4.0	1.98	0.0315
	4.5	1.841	0.041
	5.0	1.81	0.0435
	5.5	1.723	0.051
Left STG	3.0	−0.155	0.4395
	3.5	−0.301	0.3835
	4.0	−0.397	0.348
	4.5	−0.491	0.3145
	5.0	−0.553	0.2935
	5.5	−0.688	0.25
Left IPL (PGp)	3.0	1.974	0.032
	3.5	2.069	0.0265
	4.0	2.2	0.0205
	4.5	2.345	0.0155
	5.0	2.451	0.0125
	5.5	2.456	0.012
Left IPL (PGa)	3.0	1.898	0.037
	3.5	1.998	0.0305
	4.0	2.125	0.024
	4.5	2.166	0.022
	5.0	2.193	0.021
	5.5	2.145	0.023

**Figure 5:**
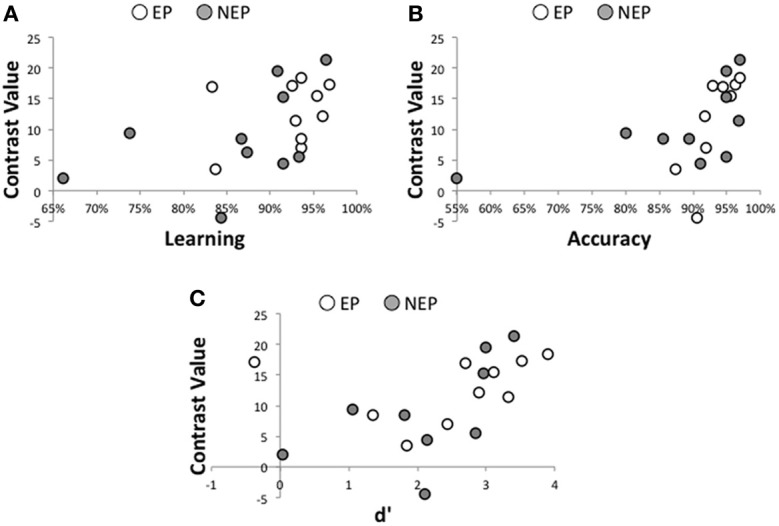
**Brain-Behavior Relationships**. For all participants, learning (measured prior to entering the scanner) is significantly related to recruitment of the left IFG while processing the newly learned language **(A)**, as is accuracy (percent correct; **B**) and discrimination sensitivity (d',**C**).

For both EP and NEP learners left IFG activity is related to behavioral performance whereas activity in the STG and AG is not. That is, the magnitude of recruitment within the left IFG while processing the newly learned language (EP or NEP > implicit baseline) is correlated with learning (average of all tests collected prior to the scan, *r* = 0.488, *p* = 0.029; Figure 5A) and performance on grammaticality judgments for the newly learned language in the scanner (percent correct: *r* = 0.507, *p* = 0.027; Figure 5B^6^; and a trend toward a relationship with d′: *r* = 0.418, *p* = 0.075; Figure 5C). These relationships were not observed in the STG (learning: *r* = 0.096, *p* = 0.687; percent correct: *r* = −0.098, *p* = 0.691 d′: *r* = −0.103, *p* = 0.674) or AG (anterior: learning: *r* = 0.169, *p* = 0.687; percent correct: *r* = −0.004, *p* = 0.986 d′: *r* = −0.146, *p* = 0.552; posterior: learning: *r* = 0.037, *p* = 0.875; percent correct: *r* = −0.116, *p* = 0.637 d′: *r* = −0.249, *p* = 0.304)^7^. Interestingly, this relationship between learning and performance in the IFG appears to be specific to the newly learned language (the MAL). The same relationship is not observed in the left IFG for making grammaticality judgments in English while processing English (percent correct: *r* = 0.155, *p* = 0.525; d′: *r* = 0.286, *p* = 0.235; reaction time: *r* = 0.194, *p* = 0.427). This was also true of the left STG (percent correct: *r* = −0.155, *p* = 0.525; d′: *r* = −0.137, *p* = 0.576; reaction time: *r* = −0.073, *p* = 0.766), left AGa (percent correct: *r* = −0.058, *p* = 0.815; d′: *r* = −0.122, *p* = 0.619; reaction time: *r* = 0.418, *p* = 0.075), and left AGp (percent correct: *r* = −0.134, *p* = 0.586; d′: *r* = −0.123, *p* = 0.615; reaction time: *r* = 0.434, *p* = 0.063). It is likely that such a brain-behavior relationship (with English) is not detectable when the language is well-established (due to ceiling effects and a lack of variability) and might be more detectable earlier in the learning process, as is observed in these data for MAL learners.

In order to localize where within the left IFG the relationship between learning and neural recruitment while processing the MAL (MAL > baseline), we entered learning scores as a regressor in the group level whole-brain analysis and found the strongest relationship in the left IFG (MNI peak coordinates: −40, 18, 12) which corresponds with the Pars Triangularis (note other relationships within the right IFG and Basal Ganglia; Table 7).

**Table 7:** **Group level whole brain regression with learning scores**.

**MAL recruitment and learning score (pre–scanner)**							
			**Coordinates (at peak)**				
**Lobe**	**Activation region**	**Hemisphere/Brodmann area**	***x***	***y***	***z***	***t*-score**	***P*-value**
Frontal	Inferior frontal gyrus	L, 45, 44	−40	18	12	3.11	0.001
	Inferior frontal gyrus	R, 45, 47	36	28	4	3.36	0.001
Sub-cortical	Caudate	L	−18	6	16	3.88	0.001
Frontal	Inferior frontal gyrus	L, 45	−42	28	4	4.35	6.80e-06

**Figure 6:**
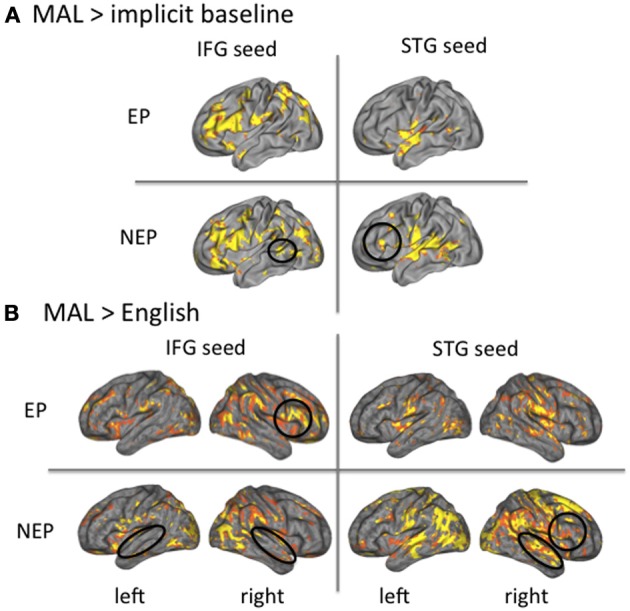
**Beta-series analysis**. The left IFG is coactive with the STG for NEP but not EP learners **(A)**. The STG and IFG are more interactive as compared to English for NEP as compared to EP learners **(B)**.

These data establish an important role of the left IFG in learning the MAL and performance, while making grammaticality judgments in the new language. Whole brain analyses also establish the importance of the STG while processing these newly learned languages, especially for NEP learners (left STG recruitment is greater for NEP than EP learners; Figure 3D). If this region is not important for making grammaticality judgments or overall learning, then why are NEP learners recruiting this region more so than EP learners? To address this question, we performed functional connectivity analyses by choosing seed regions in the left IFG and the left STG (the 10 most active contiguous, voxels within the anatomical region while processing *English* (English > implicit baseline) and searched for correlated fluctuations in activity (with the time series in the seed region: beta series analysis) the brain while individuals were processing the MAL they learned (vs. implicit baseline) (Rissman et al., 2004). First, expected beta series correlations were observed in EP and NEP learners with classic language regions in both hemispheres (Table 8). Notably, the left STG seed was coactive with the left IFG (*t* = 4.34, *p* < 0.001; Figure 6A; Table 8) and the posterior left temporal-parietal-occipital region [also important for higher-order language processing (Poeppel and Hickok, 2004), *t* = 4.44 *p* < 0.001] in NEP but not EP learners (Figure 6A; Table 8). The STG appears to be more involved in the neural network involved in processing the MAL in the NEP learners, a finding that could shed light on why NEP learners recruit this region more.

**Table 8:** **Beta series correlations**.

**Beta series correlations**							
			**Coordinates (at peak)**				
**Lobe**	**Activation region**	**Hemisphere/Brodmann area**	***x***	***y***	***z***	***t*-score**	***P*-value**
**IFG SEED, EP LEARNERS**							
Frontal	Inferior and middle frontal gyrus (location of seed)	L, 44, 45, 46, 47, 9	−50	8	12	28.32	1.58-e-20
	Inferior frontal gyrus	R, 44	54	8	8	6.09	5.64e-10
	middle frontal gyrus	L	−6	32	34	5.82	2.94e-09
Parietal	Supramarginal and angular gyrus	L, 40	−46	−52	42	4.91	4.55e-07
	Inferior parietal lobule	L, 40	−54	−30	22	4.81	7.54e-07
Occipital	Precuneus	L, 19	−32	−72	26	4.15	1.66e-06
	Precuneus	R, 19	36	−80	26	3.82	6.67-05
Other	Lentiform Nucleus	R	34	0	4	3.65	0.00013
	Lentiform Nucleus (extending to Insula)	L	−14	6	4	3.5	0.00023
**IFG SEED, NEP LEARNERS**							
Frontal	Inferior and middle frontal gyrus (location of seed)	L, 44, 45, 46, 47, 9	−50	8	12	28.26	1.58e-20
	Inferior and middle frontal gyrus	R, 44, 45, 46, 47, 13	36	16	18	4.42	4.93e-06
Parietal	Inferior parietal lobule	L, 40	−46	−52	42	4.90	4.79e-07
	Inferior parietal lobule	R, 40	44	−34	38	4.16	1.59e-05
Occipital	Precuneus	L, 19	−28	−66	28	5.00	2.86e-07
	Precuneus	R, 19	24	−68	34	4.02	2.91e-05
Other	Lentiform nucleus	L	−18	10	0	3.86	5.66e-05
	Lentiform nucleus	R	24	4	−2	3.58	0.00017
**STG SEED, EP LEARNERS**							
Temporal	Superior temporal gyrus (location of seed)	L, 22	−64	−12	4	29.10	1.58e-20
	Superior and middle temporal gyrus	R, 22	62	−16	6	6.62	1.79e-11
Frontal	Medial frontal gyrus	L, 11	−2	38	−12	3.93	4.24e-05
**STG SEED, NEP LEARNERS**							
Temporal	Superior and middle temporal gyrus (location of seed)	L, 22	−64	−12	4	29.33	1.58e-20
	Superior and middle temporal gyrus	R, 22	54	−14	−2	5.84	2.61e-09
Frontal	Inferior frontal gyrus	L, 45, 47	−48	32	0	4.34	7.12e-06
	middle frontal gyrus	L, 46	−36	42	−4	4.30	8.54e-06
	Inferior and middle frontal gyrus	R, 47, 46	40	38	−8	4.58	2.32e-06
	Medial frontal gyrus	R	2	36	−12	5.81	3.12e-09
Parietal	Angular and superior temporal gyri	L, 39	−42	−64	36	4.44	4.49e-06
	Angular gyrus	R, 39	44	−60	30	5.71	5.65e-09
Other	Hippocampus	R	34	−12	−20	4.78	8.76e-07
**MAL > ENGLISH IFG SEED, EP LEARNERS**							
Frontal	Inferior and middle frontal gyrus (location of seed)	L, 44, 45, 46, 47, 9	−50	8	12	28.87	1.58e-20
	Inferior and middle frontal gyrus	R, 45, 46, 47	50	24	10	6.12	4.68e-10
	Superior frontal gyrus	L, 10	−16	58	14	4.40	5.41e-06
**MAL > ENGLISH IFG SEED, NEP LEARNERS**							
Frontal	Inferior and middle frontal gyrus (location of seed)	L, 44, 45, 46, 47, 9	−50	8	12	28.04	1.58e-20
	Inferior and middle frontal gyrus	R, 46, 47	42	22	8	7.23	2.41e-13
Temporal	Superior and middle temporal gyrus	L	−56	2	−16	6.50	4.01e-11
	Superior temporal gyrus	R, 22	56	−2	−6	3.77	8.16e-05
Other	Lentiform nucleus	R	12	10	2	3.9	3.30e-05
**MAL > ENGLISH STG SEED, EP LEARNERS**							
Temporal	Superior and middle temporal gyrus (location of seed)	L, 22	−64	−12	4	28.39	1.58e-20
	Superior and middle temporal gyrus	R, 22	54	−10	−2	7.83	2.44e-15
**MAL > ENGLISH STG SEED, NEP LEARNERS**							
Temporal	Superior and middle temporal gyrus (location of seed)	L, 22	−64	−12	4	28.41	1.58e-20
	Superior and middle temporal gyrus	R, 22	62	0	−2	6.08	6.01e-10
Frontal	Inferior Frontal Gyrus	L, 45	−42	28	4	4.35	6.80e-06

Is this broader network recruited by NEP learners more similar to or distinct from English? To understand how networks differ from English (and thus what is more similar to native language recruitment), we conducted the same connectivity analysis (Rissman et al., 2004) in the same seed regions (IFG and STG) for a different contrast—newly learned language vs. English (MAL>English)—to reveal regions that are more co-active for processing the MAL vs. English. For EP learners, the left IFG seed was more coactive with the contralateral (right) IFG (*t* = 6.12, *p* < 0.001), and the left STG seed was also more co-active with the contralateral (right) STG (*t* = 7.83, *p* < 0.001), for MAL processing as compared to English. For the NEP learners, the left IFG seed was more co-active with the bilateral STG (left: *t* = 6.50, *p* < 0.001; right: *t* = 3.77, *p* < 0.001), and the left STG seed was more coactive both with the contralateral (right) STG (*t* = 6.08, *p* < 0.001) and ipsilateral (left) IFG (*t* = 4.35, *p* < 0.001), for MAL processing as compared to English (Figure 6B; see Table 8 for all comparisons with English). In sum, the EP network differs from English with greater recruitment of the contralateral hemisphere (both for the IFG and STG) and the NEP network differs from English with greater coactivity between the STG and IFG regions. Both connectivity profiles differ in important ways from English, with EP learners being less lateralized and NEP learners showing greater coactivity between the IFG and STG.

## Discussion

In this study, we asked whether tuning to the properties of one's native language can explain, at least in part, the sensitive period for language learning. In particular, we asked whether changing an earlier-learned (and tuned) aspect of language—sound structure—would have an impact on the neural representation of a later learned aspect—grammar. The data clearly indicate that it does. EP learners' neural recruitment overlaps more with English in key language regions (including the left IFG and left AG). Likewise, the neural circuit recruited to process the EP language is similar to the neural circuit recruited during the processing of English, albeit less lateralized (including contralateral regions). EP learners also recruit the left temporo-parietal region more than the NEP learners, a finding that could reflect greater phonetic expertise and sensory—motor integration (Buchsbaum et al., 2001). NEP learners, on the other hand, recruit the STG (bilaterally) more than EP learners. Moreover, this region appears to be part of the broader and less lateralized neural circuit used to process the NEP language that involves greater STG/IFG connectivity. We review the implications of these findings with respect to the tuning hypothesis.

Native language regions were less involved in the processing of the NEP as compared to the EP language. This was evident in the left IFG and AG, where recruitment overlapped more for English and EP than English and NEP. This pattern of findings supports our tuning hypotheses: the NEP could overlap less with English simply because cortex used for processing English is tuned for English and therefore less able to process the NEP language.

Greater recruitment of STG in NEP learners also supports the idea that native language regions are not as capable of processing the NEP language. The STG is known to be involved in phonetic processing (Hickok and Poeppel, 2000), including the perception of speech sounds (Buchsbaum et al., 2001), is engaged to a greater degree bilaterally when individuals process non-native phonological distinctions (Zhang et al., 2005), and is associated with successful learning of non-native pitch patterns in speech (Wong et al., 2007). The greater recruitment of this region for NEP learners could therefore reflect a process, whereby the brain is in the process of tuning to the sounds^8^. With more exposure to the language or perhaps more direct training on the sounds, we would expect NEP learners to recruit this region less over time.

Proficiency and fluency with language (Perani et al., 1998; Chee et al., 2002; Consonni et al., 2013) as well as cognitive demand (difficulty, more broadly construed) are important factors known to influence neural recruitment, especially in the prefrontal cortex, including the left IFG (Raichle et al., 1994; Rypma and D'Esposito, 2000; Crittenden and Duncan, 2012), both in terms of degree of recruitment (magnitude) and how the region interacts with other regions (Rypma et al., 2006; Rissman et al., 2008). Differences in recruitment across EP and NEP learners could therefore be related to these known factors. Importantly, EP and NEP learners did not differ in terms of reaction time or accuracy when assessing the grammaticality of sentences in the scanner. Likewise, we do not observe differences in the pure univariate contrast EP vs. NEP in the left PFC; rather differences are observed in degree of overlap with English and connectivity with the STG. Observed differences across languages are therefore likely to reflect requirements imposed by phonological processing and attempts to processes (and tune to) the new sounds.

While the STG appears to be involved in tuning to new sounds, recruitment of the left IFG appears to be more related to performance and learning. Indeed recruitment of the left IFG (but not the left STG) significantly correlated with performance in the scanner and, even more strikingly, learning measured prior to the scan. NEP learners' greater recruitment of STG (independently and as part of the larger language network) does not directly relate to performance. Why then are they recruiting this region so robustly? It is likely that this recruitment reflects an attempt to process (and tune to) the new sounds (Zhang et al., 2005, 2009; Wong et al., 2007).

At present, however, we cannot know for certain whether this is the case. While differences in the STG across the learning groups are especially striking, training studies such as these are expensive and limited in size (only 20 learners overall) therefore limiting the generalizability of the data. In addition, even though creating these productive MALs allows for strict control over the linguistic features of interest—both grammar and phonology—they are nonetheless still miniature and artificial. It is hard to know if differences we observe here would scale to real and larger languages. Along these lines, future research should investigate the relationship between the recruitment of the STG and IFG over time with growing phonological as well as grammatical expertise. By measuring changes in phonological expertise more directly, the “phonetic scaffold” could be characterized more fully and the influence of this learning on grammar learning (both behaviorally and in the brain) could be much better understood. Exposure is also likely to impact learning outcomes. It could be (and is very likely) that 4 days of exposure to novel phonology is not nearly enough to build the phonemic maps necessary to process new sounds, but increased exposure would result in overcoming this and developing the requisite “scaffolding.” Delays in the making of this scaffold are likely to be part of the cause of adult language-learning difficulties and further work needs to characterize this alongside grammatical learning during longer periods of time in adults.

Further work characterizing the anatomical and functional specificity of these scaffolds is also necessary. Much recent work aims to characterize the functional specificity of sub-regions both within in the IFG (Fiebach et al., 2006; Fedorenko et al., 2011) and the STG (Indefrey and Levelt, 2004) and to more carefully specify the functional anatomy of language (Poeppel and Hickok, 2004). While this is not possible in the current sample (functional localizers were not employed and the sample is insufficient for extensive brain-behavior analyses), it should be an important goal of future investigation especially for thinking about possible learning interventions.

Despite the need for further studies, our findings have implications for understanding the sensitive period for language learning. Neural recruitment—even when proficiency is matched—differs across EP and NEP learners. The ways in which this recruitment is different (additional STG, less overlap with English in the left IFG) is consistent with the nested tuning theory which predicts that differences in more foundational aspects of language (such as sounds) should have implications for the neural representation of aspects of language that depend on the foundational ones (grammar). We show that it does. Adults' difficulty in learning language may therefore be due to the recruitment of the “wrong” neural scaffolding.

## Author contributions

Amy S. Finn and Carla L. Hudson Kam developed the idea for the study. Marc Ettlinger and Mark D'Esposito contributed to the study design. Testing and data collection were performed by Amy S. Finn. Amy S. Finn, Jason Vytlacil, and Marc Ettlinger performed the data analysis and interpretation under the supervision of Carla L. Hudson Kam and Mark D'Esposito. Amy S. Finn drafted the paper, and all co-authors provided critical revisions. All authors approved the final version of the paper for submission.

### Conflict of interest statement

The authors declare that the research was conducted in the absence of any commercial or financial relationships that could be construed as a potential conflict of interest.

## Footnotes

^1^It is well-established that these very process that increase during childhood—executive function and memory—also decrease with aging. See Luo and Craik (2008), for a full review.

^2^Following these constraints, 60 possible words were actually generated, of which 30 were chosen based on English-likeness ratings from native English speaking raters blind to the overall goals of the study (*n* = 10).

^3^One hundred and fifty phones that do not occur in English were chosen from a list of phonemes from across the world's languages (Maddieson, 1984). Native English speaking participants blind to the study design rated these phones, presented individually, on their English-likeness (*n* = 10). The lowest ranked phones (13 vowels, 19 consonants) were chosen for constructing the words.

^4^The results of earlier tests (not end-point tests presented here) are the subject of another paper currently in preparation. For the purposes of measuring neural activation, we were focused on equating for proficiency prior to scanning.

^5^While vocabulary performance differs across learning groups both overall [across all days tested: *t*_(18)_ = 3.130, *p* = 0.006] and during the final test day [*t*_(18)_ = 2.33, *p* = 0.032], it is very high for both EP (mean = 98.6% correct, *SD* = 0.023) and NEP (mean = 93.7% correct, *SD* = 0.064).

^6^Notice there is one statistical outlier who has very low accuracy (55%). This subject's performance was also low on grammaticality judgments in English (60%) and so this low performance is likely due to factors other than not learning the new language. Only correct trials were included in the brain analyses and this brain-behavior correlation remains significant when this outlier is excluded (percent correct: *r* = 0.523, *p* = 0.026).

^7^Note that these relationships are only marginally significant (between learning and recruitment of the IFG and percent correct and recruitment of the IFG) when corrections for multiple comparisons are made (bonferroni *p* for 3 tests per *DV* = 0.017).

^8^The STG is of course not the only region in the brain that is associated with phonological processing. In fact, prefrontal regions (the IFG) are associated with phonological decoding and processing and the Medial Temporal Gyrus (MTG) is also widely implicated along with more posterior superior temporal regions [See Poeppel and Hickok (2004), for a comprehensive review]. Likewise, successful learning of non-native contrasts is associated with recruitment of the same regions used for native contrasts: the left STG, the insula (frontal operculum), and left IFG (Golestani and Zatorre, 2004).
